# Recurrent mantle cell lymphoma in the uterine cervix: a case report

**DOI:** 10.1186/s13256-020-02487-6

**Published:** 2020-09-02

**Authors:** Giusi Santangelo, Innocenza Palaia, Giorgia Perniola, Anna Di Pinto, Angela Musella, Federica Tomao, Margherita Fischetti, Carolina Sassu, Mariagrazia Piccioni, Violante Di Donato, Pierluigi Benedetti Panici

**Affiliations:** Department of Maternal and Child Health and Urological Sciences, Policlinico “Umberto I”, University “Sapienza”, Viale del Policlinico, 155, 00161 Rome, Italy

**Keywords:** Aggressive non-Hodgkin lymphoma, Cervix, Mantle cell lymphoma, Quality of life, Relapse, Postmenopausal vaginal bleeding

## Abstract

**Background:**

Mantle cell lymphoma is one of several subtypes of non-Hodgkin lymphoma. Cervical relapse of non-Hodgkin lymphoma is a very rare condition that has a variable and nonspecific presentation and may resemble other neoplastic or inflammatory conditions.

**Case presentation:**

Our patient was a 58-year-old Caucasian woman who experienced relapse of mantle cell lymphoma with cervical localization. She complained of postmenopausal vaginal bleeding, bladder pressure, and rapid growth of a cervical lesion. An irregular tumor mass of the cervix was visualized during gynecological examination, with findings highly suggestive of locally advanced cervical cancer. Surprisingly, the biopsies showed an extra nodal relapse of mantle cell lymphoma in the cervix. The rarity of this presentation and the scarcity of clinical studies make this type of recurrence very aggressive and difficult to treat.

**Conclusions:**

Obtaining a definitive histological diagnosis by biopsy or surgical resection and starting appropriate therapy are essential for recovery and treatment of these patients, even if the prognosis is poor.

## Introduction

Mantle cell lymphoma (MCL) is one of several subtypes of non-Hodgkin lymphoma (NHL). MCL is the rarest of the subtypes, accounting for about 6% of all NHL cases in the United States and Europe. It is the result of a malignant transformation of a B lymphocyte in the outer edge of a lymph node follicle, called the “mantle zone.” Those cells can spread through the lymphatics and blood to other lymph nodes or tissues, such as the bone marrow, liver, and gastrointestinal tract. MCL has the worst prognosis among lymphomas, with a median survival of approximately 3–4 years [1].

Lymphoid neoplasms of the female genital organs are relatively rare, accounting for less than 5% of extranodal lymphomas. We conducted a literature search of PubMed and MEDLINE using the keywords “cervix,” “mantle cell lymphoma,” and “lymphoma.” We found no reports of MCL involving the uterine cervix. We present a case of a patient with a cervical relapse of NHL with a rare presentation. This condition is very rare and, to our knowledge, has never been described in the literature. The aim of this case report is to remind clinicians to think of cervical relapse of NHL in cases of vaginal bleeding in women with a history of NHL.

## Case presentation

A 58-year-old Caucasian woman complaining vaginal bleeding and a cervical mass was admitted to our hospital. She reported menopause at 52 years. She had a history of mantle cell NHL. She had no comorbidity, did not take drugs, denied smoking and the use of alcohol, and denied any family history of cardiovascular diseases and neoplasia. The result of her neurological examination was negative. Clinically, the patient was in good general condition and reported weight loss in the last year following her diagnosis of NHL. At admission, her vital signs were good; her pulse was 76 beats/minute, her blood pressure was 120/80 mmHg, and her body temperature was 36.5 °C.

**Fig. 1:**
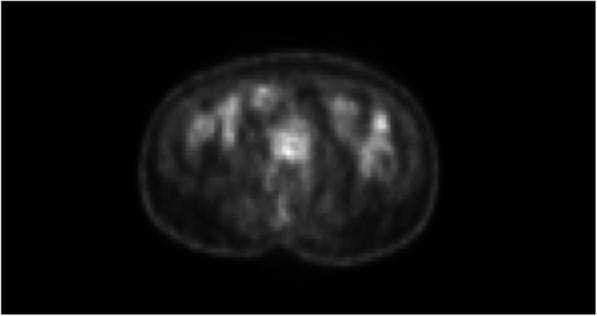
Positron emission tomographic images

**Fig. 2:**
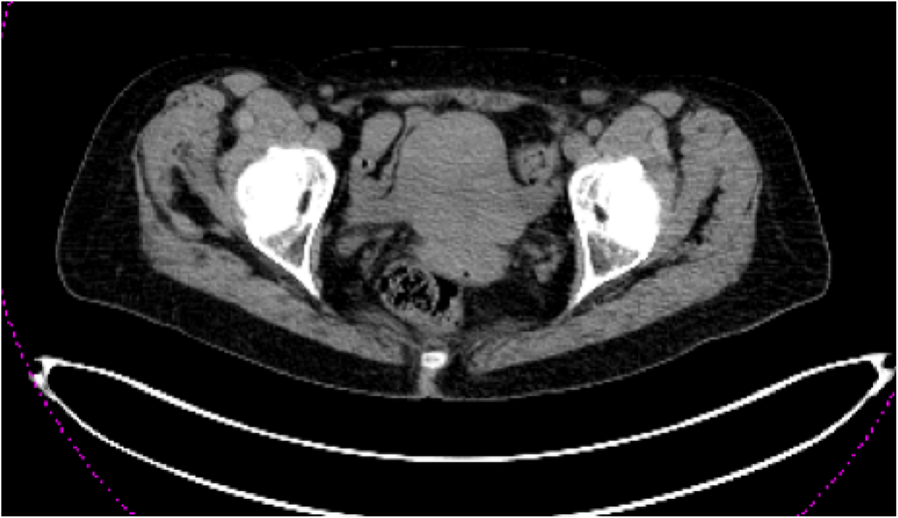
Computed tomographic scan

Briefly, in March 2018, she presented with right groin and thoracic lymph node involvement (stage IIIa). Treatment consisted of chemotherapy with R-CHOP (rituximab, cyclophosphamide, doxorubicin, vincristine, prednisone) and R-DHAP (rituximab, cisplatin, cytarabine, dexamethasone) for six cycles, alternating. For central nervous system prophylaxis, four lumbar punctures with methotrexate were performed. In October 2018, she received chemotherapy with FEAM (fotemustine, cytarabine, etoposide, melphalan) sequenced by infusion of autologous peripheral stem cells. The transplant was complicated by septic shock and atrial fibrillation. In November 2018, the patient was discharged in good general condition. She had regular negative follow-up results until January 2019, when positron emission tomography–computed tomography (CT) showed a hypercaptation at the level of the uterine cervix (maximum standardized uptake value 11.8) (Figs. 1 and 2).

In April 2019, the patient was admitted to our department for a gynecological examination. She complained of postmenopausal vaginal bleeding in the last 2 months, bladder pressure, and rapid growth of a cervical lesion.

**Fig. 3:**
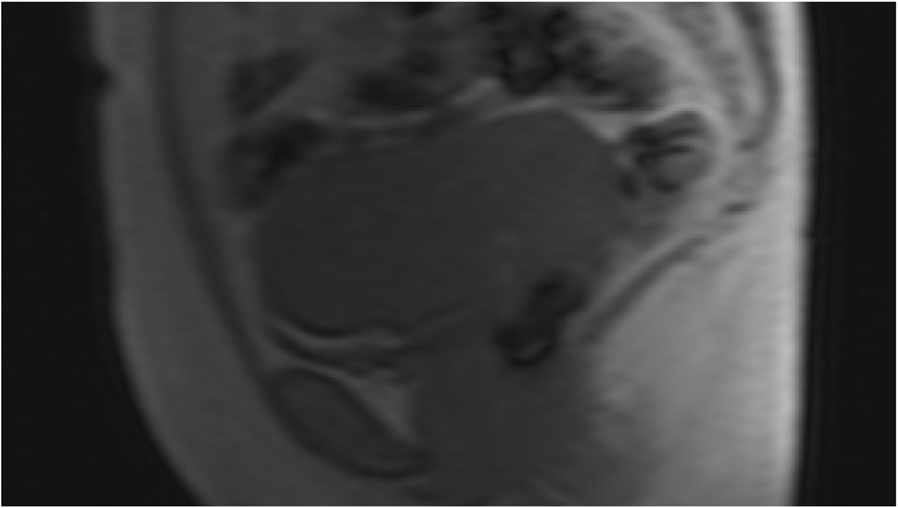
Magnetic resonance imaging studies

An irregular tumor mass of the cervix was visualized during gynecological examination, highly suggestive of locally advanced cervical cancer. However, the surface appeared smooth with no exophytic or erosive components, and the lesion was measured as 5 cm in diameter. The disease extended into the right paracervical and parametrial tissue, invading the proximal two-thirds of the vaginal submucosa. Ultrasound examination showed a 5-cm highly vascularized cervical tumor extending into the right parametrium and bladder without mucosal infiltration. Magnetic resonance imaging (MRI) was performed, showing a locally invasive tumor with heterogeneous high-intensity signaling on T2-weighted images, most likely originating from the cervix, measuring 50 × 33 mm (Fig. 3). Invasion into one-third superior of the vagina, bladder, and bilateral parametria was visualized. The CT scan showed a similar extent of cervical tumor. No enlarged lymph nodes or lesions in the liver, lung, or bone were visualized.

Surprisingly, the biopsies revealed a neoplastic cell with a positive test result for CD20 and Ki67 100% and negative test results for CD3, CD5, CD10, BCL2, S100, and cytokeratin. These findings confirmed the diagnosis of a relapse of MCL with cervical localization.

She was referred to the hematology department and was started on ibrutinib 560 mg/day from July 2019 to October 2019. Transvaginal ultrasound in September 2019 documented persistence and augmented volume (41 × 30 × 33 mm) of the cervical lesion.

The patient is still under treatment with ibrutinib, but a whole-body CT scan showed persistence of disease, and her general condition is poor, with severe asthenia and loss of hearing.

## Discussion

Our patient presented with vaginal bleeding; in menopause, this sign is suspicious for uterine (endometrial and cervical) neoplasia. At admission, the first suspicion was of cervical neoplasia, despite the NHL history of our patient. The clinical examination did not drive the clinicians through the differential diagnosis, because the follow-up results had been negative until that moment. In the literature, there are no studies that have described cervical relapse of NHL, so clinicians must keep cervical relapse of NHL in mind in cases of vaginal bleeding in women with a history of NHL.

MCL is a relatively uncommon subtype of lymphoid malignancy and represents 5–7% of malignant lymphoma in Western Europe. The annual incidence of this disease has increased during recent decades to 1–2 per 100,000.

MCL is more common in men than in women, with a 3:1 ratio [2]. Extranodal involvement of NHL is a common finding. However, only 10–35% of NHL cases present with primary extranodal NHL. Female genital tract involvement can in rare cases be a site of origin [3]. Extranodal lymphoma occurs in approximately 40% of all patients with lymphoma; extranodal disease is more common with NHL. It causes many deaths worldwide, and its incidence is increasing. It represents a heterogeneous group of neoplasms originating from lymphocytes. Diffuse large B-cell lymphoma is the most common histological NHL subtype in adult patients [4].

The majority of relapses in NHL occur in the first 2 years after the completion of treatment. The majority of relapses are symptomatic, and they are rarely identified by surveillance imaging alone. Extranodal involvement often appears in the gastrointestinal system, followed by skin (10% of cases) [5].

In 1972, Freeman *et al.* reported a case series of 1467 extranodular lymphomas, of which only 3 (0.2%) were found to originate in the cervix [6]. A publication in 1995 stated that 18 (0.6%) of 2733 NHL cases originated from the uterus, cervix, or paracolpos [7]. Lymphoma accounts for only 0.3% of all cervical cancers, making it a rare malignancy. In recent decades, the incidence of extranodal NHL has increased. The hypothesized etiology of the increase includes immunosuppressive treatments, human immunodeficiency virus/acquired immunodeficiency syndrome-related immunosuppression, environmental exposure to pesticides, and improved diagnostic techniques [8].

Due to the rarity of extranodal cervical NHL, no prospective randomized trials exist to evaluate diagnostic tools or treatment options. The review below is therefore based mostly on case reports and small case series. Pelvic ultrasound and CT are useful in the diagnosis and staging of cervical malignancies. However, MRI is the most effective method for imaging evaluation of the cervix. MRI could possibly be used to differentiate cervical and uterine lymphomas from other entities. Cervical NHL is best defined on the basis of T2-weighted images or contrast-enhanced T1-weighted images. Suggestive MRI findings for lymphomas include an isointense or hypointense signal relative to the myometrium on T1-weighted images, relatively homogeneous high-intensity signaling on T2-weighted images, and lack of clear margination with moderate uniform enhancement [9].

For the same reason, there is no consensus in the literature concerning the optimal management of cervical extranodal NHL. Treatment regimens of cervical extranodal NHL have been reported in several case reports and case series, including surgery, chemotherapy, radiotherapy, or a combination of two or three treatments.

## Conclusion

Cervical relapse of NHL is a very rare condition and has a variable and nonspecific presentation, and it may resemble other neoplastic or inflammatory conditions. Obtaining a definitive histological diagnosis by biopsy or surgical resection and starting appropriate therapy are essential for recovery and long-term survival of these patients, in whom the prognosis is poor.

## Data Availability

Not applicable.
